# A Solution of Salicylic Acid

**Published:** 1875-12

**Authors:** F. Alfred Reichardt

**Affiliations:** Physician and Pharmacist


A Solution of Salicylic Acid.—*By F. Alfred Reichardt.*—The most concentrated solution of Salicylic Acid has been, up to the present, obtained by using borax, phosphate of soda, and phosphate of ammonia, but as these will only dissolve about one-third of their weight, in about seventeen times their bulk of water, we only obtain a solution of ten grains to the ounce.

After repeated experiments, I have found that thirty grains of salicylic acid, with one drachm of saturate of ammonia, and water enough to make one fluid ounce, will give a clear solution. This is, I belive, the strongest solution of free salicylic acid known, and the solvent used has never been mentioned in any journals here or in Europe.

In preparations where salicylic acid is intended for external use, a German writer reports that a solution of it in sulphite of soda, is of the greatest benefit. The antiseptic properties of the salicylic acid are much enhanced by this addition, and it is stated that the application is not painful to wounds or excreted surfaces. The formula is as follows:

| | |
|---|---|
| Salicylic Acid | 10 grains. |
| Sulphite of Soda | 30 grains. |
| Water | 1 fl. oz. |

It has, to my knowledge, been used with great benefit in several cases of eczema.—*Physician and Pharmacist.*

